# Deep Learning–driven classification of external DICOM studies for PACS archiving

**DOI:** 10.1007/s00330-022-08926-w

**Published:** 2022-07-05

**Authors:** Frederic Jonske, Maximilian Dederichs, Moon-Sung Kim, Julius Keyl, Jan Egger, Lale Umutlu, Michael Forsting, Felix Nensa, Jens Kleesiek

**Affiliations:** 1 Institute of AI in Medicine (IKIM), University Hospital Essen, Girardetstraße 2, 45131 Essen, Germany; 2 Cancer Research Center Cologne Essen (CCCE), University Medicine Essen, Essen, Germany; 3 Institute of Diagnostic and Interventional Radiology and Neuroradiology, University Hospital Essen, Essen, Germany; 4 Department of Tumor Research, University Hospital Essen, Essen, Germany; 5 German Cancer Consortium (DKTK), Partner Site Essen, Essen, Germany; 6 University Duisburg-Essen, Essen, Germany

**Keywords:** Artificial intelligence, Machine learning, Radiology information systems

## Abstract

**Objectives:**

Over the course of their treatment, patients often switch hospitals, requiring staff at the new hospital to import external imaging studies to their local database. In this study, the authors present MOdality Mapping and Orchestration (MOMO), a Deep Learning–based approach to automate this mapping process by combining metadata analysis and a neural network ensemble.

**Methods:**

A set of 11,934 imaging series with existing anatomical labels was retrieved from the PACS database of the local hospital to train an ensemble of neural networks (DenseNet-161 and ResNet-152), which process radiological images and predict the type of study they belong to. We developed an algorithm that automatically extracts relevant metadata from imaging studies, regardless of their structure, and combines it with the neural network ensemble, forming a powerful classifier. A set of 843 anonymized external studies from 321 hospitals was hand-labeled to assess performance. We tested several variations of this algorithm.

**Results:**

MOMO achieves 92.71% accuracy and 2.63% minor errors (at 99.29% predictive power) on the external study classification task, outperforming both a commercial product (82.86% accuracy, 1.36% minor errors, 96.20% predictive power) and a pure neural network ensemble (72.69% accuracy, 10.3% minor errors, 99.05% predictive power) performing the same task. We find that the highest performance is achieved by an algorithm that combines all information into one vote-based classifier.

**Conclusion:**

Deep Learning combined with metadata matching is a promising and flexible approach for the automated classification of external DICOM studies for PACS archiving.

**Key Points:**

*• The algorithm can successfully identify 76 medical study types across seven modalities (CT, X-ray angiography, radiographs, MRI, PET (+CT/MRI), ultrasound, and mammograms).*

*• The algorithm outperforms a commercial product performing the same task by a significant margin (> 9% accuracy gain).*

*• The performance of the algorithm increases through the application of Deep Learning techniques.*

**Supplementary Information:**

The online version contains supplementary material available at 10.1007/s00330-022-08926-w.

## Introduction

Machine learning has had a large impact on the field of medicine, particularly in radiology [1]. Great strides have been made in the automated assessment of medical images in decision-making [2], prediction [3], and diagnostics [4]. An often-overlooked application of Deep Learning is the elimination of repetitive tasks in clinical routines. One of these tasks, currently requiring dedicated medical-technical personnel, is the processing and archiving of external DICOM studies. Frequently, patients from other facilities submit their imaging studies, which are then archived in the local hospital PACS. The facility of origin of these studies occasionally has a different language, a different naming standard for procedures, differently composed imaging studies, or even different procedures. Adherence to DICOM standards, such as clean labeling, file structure, and ordering (see for example [5]), is not always guaranteed, and Güld et al [6] have found that DICOM metadata is often unreliable (the DICOM tag *Body Part Examined* was found to be incorrect in 15.3% of cases). Studies that are thus incorrectly mapped to the local study nomenclature cause problems, from medical staff being unable to identify the relevant study for diagnostics and treatment, to arranging for a procedure to be performed unnecessarily. The lack of adherence to established standards additionally gives rise to a compositionality problem—a single study can contain multiple different series which together comprise one class, and such a composition can be incompatible with the classification scheme of the recipient hospital.

Related works have performed body region classification using various Deep Learning strategies (see [7–11]), but none with the explicit goal of study classification for PACS archiving in mind, nor for this comprehensive list of modalities.

In contrast to Dratsch et al [7], who used a neural network for evaluation of radiographs in the same context, the authors of this paper propose to extend the scope of the application to multiple modalities simultaneously—CT, X-ray angiography, radiographs, MRI, PET, ultrasound, and mammograms. Firstly, we aim to provide an automated classification algorithm, which can be integrated in the clinical routine. Secondly, we aim to establish the usefulness of neural networks in this context, as a part of or as a standalone solution. In this study, we propose a Deep Learning–based approach and compare its performance to a commercial product.

## Materials and methods

### Training images

For this retrospective study, we retrieved 11,934 de-identified imaging series belonging to 4597 separate examinations from the local PACS. These imaging series covered random timeframes and patients, and originated from 76 different types of studies. We acquired around 150–200 imaging series for each class, or fewer if unavailable. These classes (see Supplementary Materials) comprise the most common study types at our hospital. Imaging series were automatically labeled according to the class they were saved as in the PACS. Images were only rejected based on their series descriptor (excluding non-representative series such as topograms), but not quality or demographics. This is justified, as the external test set might feature low-quality scans and previously unseen image compositions. Ten percent (each) of the images are randomly drawn from the internal dataset to create a validation and test set for performance evaluation.

### External studies

We collected 843 external studies (from 522 patients arriving at the local hospital from January 4, 2021, to January 8, 2021), which were labeled by a radiographer of the medical-technical staff with several years of experience as a team leader. Every study received at least one label, and some studies received several labels if they could be reasonably interpreted as multiple study types. This external test set was not used during training or for fine-tuning. Accuracy and predictive power on this set yield the final performance measures. A breakdown of the origins of the external studies can be found in Table 1.

**Table 1:** Origin of external studies. A breakdown of the origin of the external imaging studies used in this work. Local studies refer to studies from the University Hospital Essen. Regional studies refer to studies from the federal county of Northrhine-Westphalia. National studies originated anywhere in Germany. The international studies originated in hospitals situated in the Netherlands (3 in 2 facilities), Belgium (1), Austria (2), and Romania (1). The language used sometimes varied between the DICOM tags (presumably because some information is automatically filled in). Typically, most metadata available was in the language of origin country while some of it was in English. Some studies had empty or uninformative entries for their origin (such as “Hospital”)

External images	Imaging studies	Hospitals
Local	150	30
Regional	584	238
National	70	48
International	7	5
Unknown	32	N/A
**Sum**	**843**	**≥ 321**

In order to establish a measure of interrater reliability, another rater, a second-year resident in oncology with experience in evaluation of radiological images, labeled a subset of 300 of the external studies. The second rater was blinded to the labels of the first rater. Labels were considered to be in agreement if at least one of the labels provided by both raters matched.

The anonymization was performed using DicomDeidentify. A full list of DICOM tags that are erased or set to a common default value is provided by the authors in their code repository (https://github.com/luckfamousa/DicomDeidentify).

At our institution, imaging studies follow a specific structure (one study folder containing several series folders, each containing only the DICOM files of its respective series) and the DICOM tag entries, including the procedure names, are standardized. For external studies, this is often not the case, and neither standard is guaranteed. Thus, our acquisition of metadata is programmed to be agnostic to the structure of the imaging study, and we always evaluate several different tags.

### Image preprocessing and neural network training

**Fig. 1:**
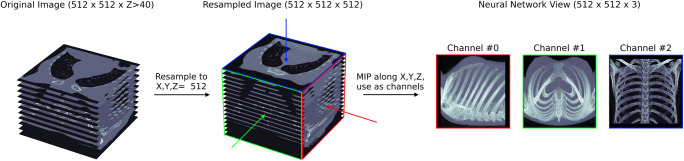
Image preprocessing visualization. Graphical representation of an example thoracic CT. The imaging series is recognized as a 3D series and resampled into cubic shape. The MIPs along the major axes are combined into the different channels of one image, which the neural network can use for training

We evaluated two neural networks, a Resnet-152 [12] and DenseNet-161 [13], both pre-trained on ImageNet [14]. Images fed into the networks were first normalized into the range [0,1]. All image series were reduced to single-channel, greyscale images, and resampled to 512 × 512 along the *X*- and *Y*-axis, using spline interpolation. Two-dimensional image series became 512 × 512 × 3 images by stacking the first, middle, and last layer along the *Z*-axis. If only a single image was in the series (e.g., a single sonographic image), the same image was reused for all three channels. Three-dimensional images were resampled to length 512 along the *Z*-axis. The 512 × 512 × 3 input images were created by constructing the maximum intensity projections (MIPs) along the major axes. To avoid resampling errors, series where *Z* < 40 were treated as two-dimensional. Typical examples for two-dimensional images in this context are single sonographic images or a set of radiographic images from different perspectives. Typical examples for three-dimensional images are high-resolution CT or MRI image series. The process is depicted in Fig. 1.

If an image had a time axis, either it was treated as a two-dimensional image series (for 2D+T images), and the 512 × 512 × 3 image was created by stacking the first, middle, and last time step, or it was sampled at the halfway point in time and then treated like a regular three-dimensional image (for 3D+T images).

Images were randomly flipped or rotated during training (with probability *p* = 0.2), as image orientation can vary on external images. Performance was evaluated using cross-entropy loss. The networks were trained using transfer learning, freezing every layer except the final layer and the classifier (for hyperparameters, see Supplementary Materials). To eliminate unnecessary cross-contamination, we created an independent network for every modality except PET. For PET CT/MRI studies, the CT/MRI images were evaluated by the respective network. The PET images themselves were not used during training or inference.

Additionally, temperature scaling was performed using the hold-out validation set, so the network confidences become statistically meaningful probabilities [15]. This has a positive effect on study prediction accuracy (see Supplementary Materials).

### MOMO

**Fig. 2:**
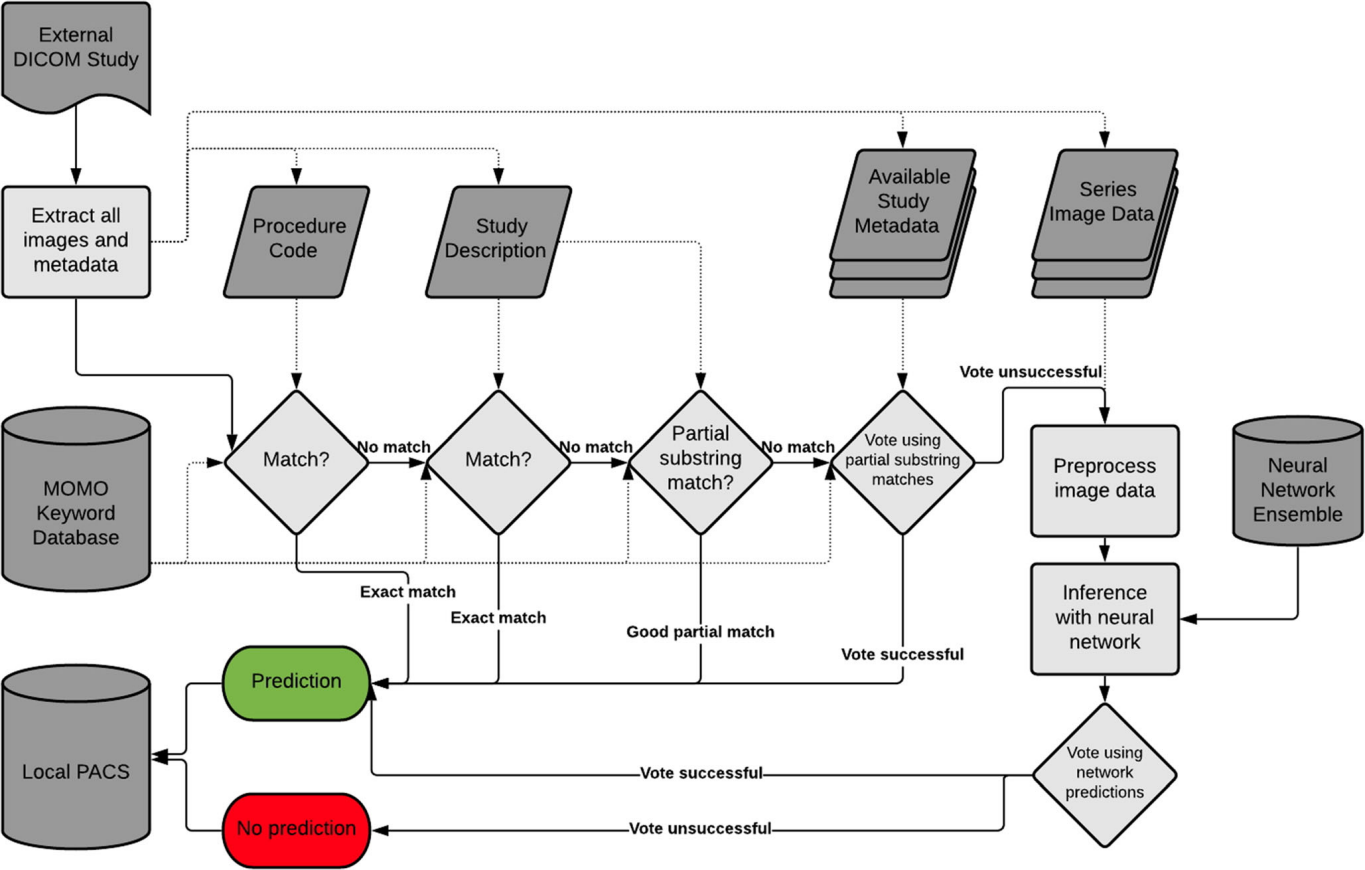
MOMO structure. MOMO extracts meta- and imaging data and then progresses through its layers one by one until a decision is reached. The voting approach used for combining information is detailed in the “Methods” section. The entire algorithm can be modified from the Modality Mapping Database (a single tabular reference document containing all study classes and associated keywords), and a single configuration file (which holds technical parameters). This figure depicts one specific configuration of MOMO (the default configuration). It corresponds to the first algorithm in Table 2, labeled “NWE in layer 5,” as the network ensemble is the fifth resource to be used

The MOdality Mapping and Orchestration’s (MOMO) algorithm follows a hierarchical layer structure (see Fig. 2). During each step, MOMO attempts a prediction. If successful, it returns the prediction, else it proceeds to the next step. In the default configuration, it begins by matching the *Procedure Code* (DICOM tag) of the study against known items. Next, the *Study Description* is evaluated. If both fail, it attempts to partially match the *Study Description*. After that, it extracts all pre-specified metadata keys from every series in the study and attempts to match these against a list of pre-specified keywords. Each match is weighted equally, with a simple majority choosing the prediction. A rules system disallows or modifies some votes based on a configuration file (see Supplementary Materials). In case of a tie, or if no votes were given, all imaging series are resampled and fed into the neural network corresponding to their modality. Each network prediction yields one vote, weighted by the confidence of the prediction. All votes are summed and evaluated.

We designed and evaluated a small Monte Carlo experiment as a proof of concept for this voting approach in an idealized setting (see Supplementary Materials).

The code, including instructions and a publicly available test case, is available from https://github.com/TIO-IKIM/MOMO_Submission_Code.

### Statistical analysis

To assess the performance of the neural networks on a per-series basis, its accuracy is calculated for the internal test set, using fivefold cross-validation. Accuracies are reported with 95% confidence intervals.

MOMO is evaluated on the external dataset and compared to the Pan-Importer [16], a commercial product performing the classification task at our facility. An ablation study is performed, where the decision power of the network is increased by moving the network higher in the decision hierarchy (see Table 2). For every algorithm, we report the percentage of correct predictions, minor and major errors, accuracy, and network contribution, on the external test set. An error is considered minor if the classification is correct but too general (e.g., predicting “MR Spine” instead of “MR Thoracic Spine”), since this will not cause unnecessary examinations to be performed or studies not to be found. Additionally, we report which part of each algorithm was responsible for how many predictions.

**Table 2:** Breakdown of MOMO algorithm variations. A breakdown of the different algorithms that we test and report results for in the “Results” section. In every layer, MOMO attempts to make a prediction using a specific resource. If it can, it exits and returns the prediction. If not, it proceeds to the next layer. We vary the decision power of the network by moving it into an increasingly high layer. Additionally, we test a variation without neural network support and two variations in which we merge some layers

Algorithm description	Layer 1	Layer 2	Layer 3	Layer 4	Layer 5
NWE in layer 5	Procedure code, exact match	Study description, exact match	Study description, partial match	Other DICOM tags, partial matches, decided by vote	Neural network ensemble, decided by vote
NWE in layer 4	Procedure code, exact match	Study description, exact match	Study description, partial match	Neural network ensemble, decided by vote	Other DICOM tags, partial matches, decided by vote
NWE in layer 3	Procedure code, exact match	Study description, exact match	Neural network ensemble, decided by vote	Study description, partial match	Other DICOM tags, partial matches, decided by vote
NWE in layer 2	Procedure code, exact match	Neural network ensemble, decided by vote	Study description, exact match	Study description, partial match	Other DICOM tags, partial matches, decided by vote
No neural networks	Procedure code, exact match	Study description, exact match	Study description, partial match	Other DICOM tags, partial matches, decided by vote	
Pure NWE	Neural network ensemble, decided by vote				
Merged (L4 + NWE)	Procedure code, exact match	Study description, exact match	Study description, partial match	Other DICOM tags, partial matches + neural network ensemble, combined vote	
Merged (L3 + L4 + NWE)	Procedure code, exact match	Study description, exact match	Partial matches for study description + Partial matches for other DICOM tags + Neural network ensemble, combined vote		

A breakdown of the layer structure for the variations of our algorithm reported in the results section can be found in Table 2.

## Results

We justified the MOMO voting approach (Fig. 1) in a Monte Carlo experiment (see Supplementary Material), showing that study-class prediction accuracy non-linearly increases with an increasing number of series in a study.

We evaluated multiple neural networks for different imaging modalities. The accuracies for the networks are shown in Table 3 and compared to the state of the art (SOTA), if available. We achieved comparable performance across all modalities. Conventional radiographs achieved the highest prediction accuracy (97.1%, CI: 96.2–98.0%), outperforming the current SOTA [7], and ultrasounds the lowest (81.4%, CI: 76.7–86.1%).

**Table 3:** Network training results. Per-series classification accuracy of the neural networks. Accuracy and 95% confidence interval (CI) are reported. If applicable, a comparison with the best-known comparable literature value is made (note that these have different underlying scopes, training, and test sets). All our results are derived from fivefold cross-validation. Networks marked with a (*) symbol are kept for MOMO

Modality	ResNet-152	DenseNet-161	SOTA in literature
CR	96.5% (CI: 95.0–98.0%)	97.1% (CI: 96.2–98.0%)*	90.3% (CI: 89.2–91.3%)
CT	84.6% (CI: 81.7–87.5%)	87.7% (CI: 85.2–90.2%)*	**91.9% (CI: 90.2**–**92.1%)**
MRI	80.2% (CI: 77.4–82.9%)	85.4% (CI: 83.4–87.5%)*	**94.2% (CI: 92.0**–**95.6%)**
US	79.0% (CI: 75.0–83.0%)	**81.4% (CI: 76.7**–86.1%)*	–
XA	**83.5% (CI: 77.3**–89.6%)*	81.2% (CI: 76.6–85.9%)	–

**Fig. 3:**
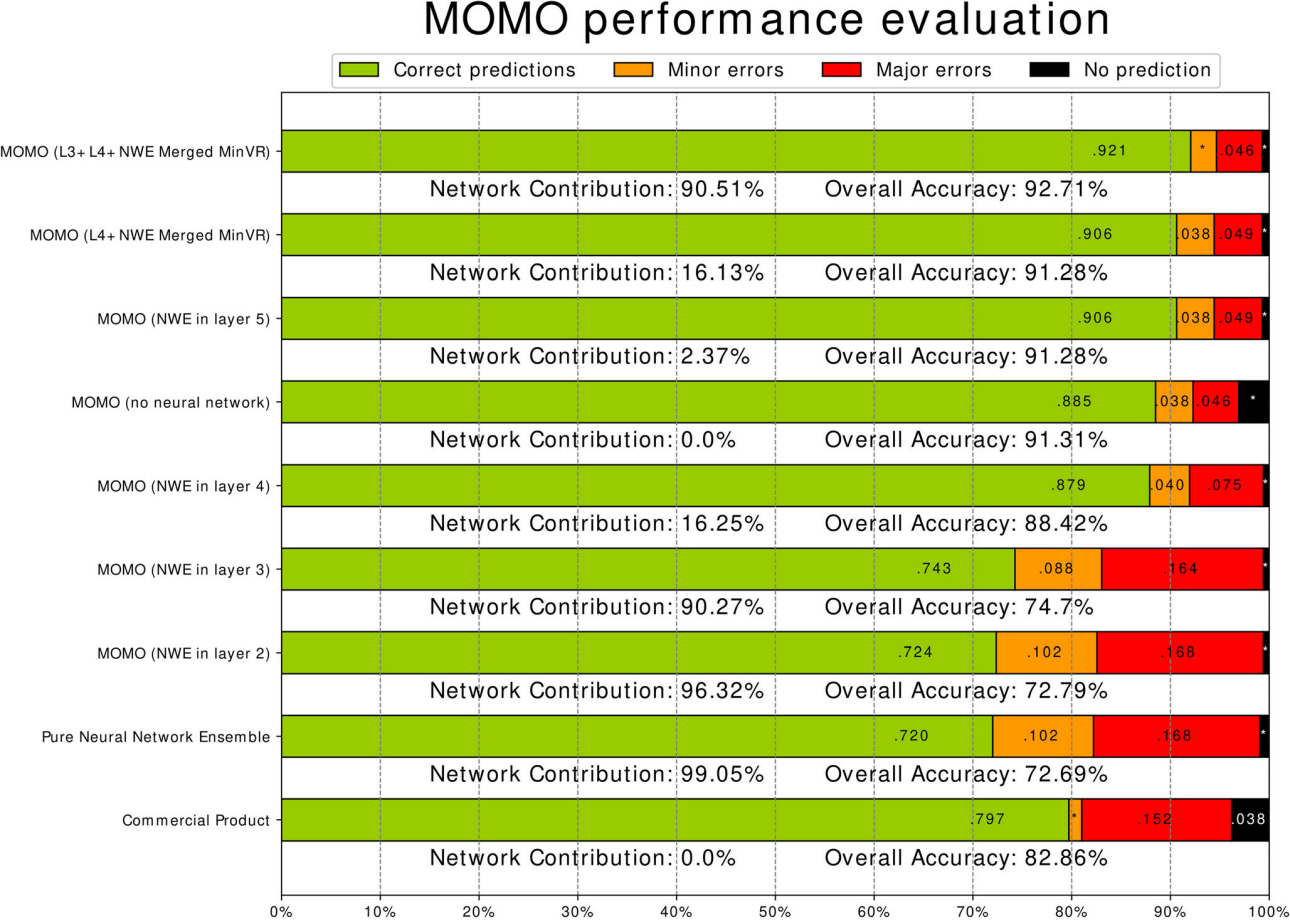
Performance evaluation. A stacked bar plot, displaying correct predictions, minor and major errors for different study classifiers. Included are MOMO with and without network ensemble (NWE) with varying decision power (refer to Table 2 for details), as well as a classifier based purely on neural networks and the commercial product. Additionally, two hybrid variants, where the network votes together with other layers (merged votes), are tested. On the “Minimal Vote Rules” (MinVR) setting, fewer rules for vote modification (see the “MOMO” section) were applied for technical reasons. We report classification accuracy, number of correct, and incorrect predictions (split by severity of error) and the contribution of the networks (if any)

We also explored the study classification performance of MOMO, testing a wide range of settings and methods to combine metadata and image information. The results of the performance evaluation can be seen in Fig. 3. The network ensemble alone achieved 99.05% predictive power, 72.69% accuracy, and 10.3% minor errors. The commercial product reached 96.20% predictive power, 82.86% accuracy, and 1.36% minor errors.

Overall, we found that a hybrid version of MOMO yielded the best performance, utilizing all metadata and neural networks in a single large vote. It outperformed other variants by a significant margin, scoring 99.29% predictive power, 92.71% accuracy, and 2.61% minor errors (using the networks with the best cross-validation performance).

Given the agreement criterion described in the “External studies” section, interrater agreement was 96.3% with a Cohen’s kappa of *κ* = 0.958. This value can be considered an approximate baseline for human performance on this task.

**Fig. 4:**
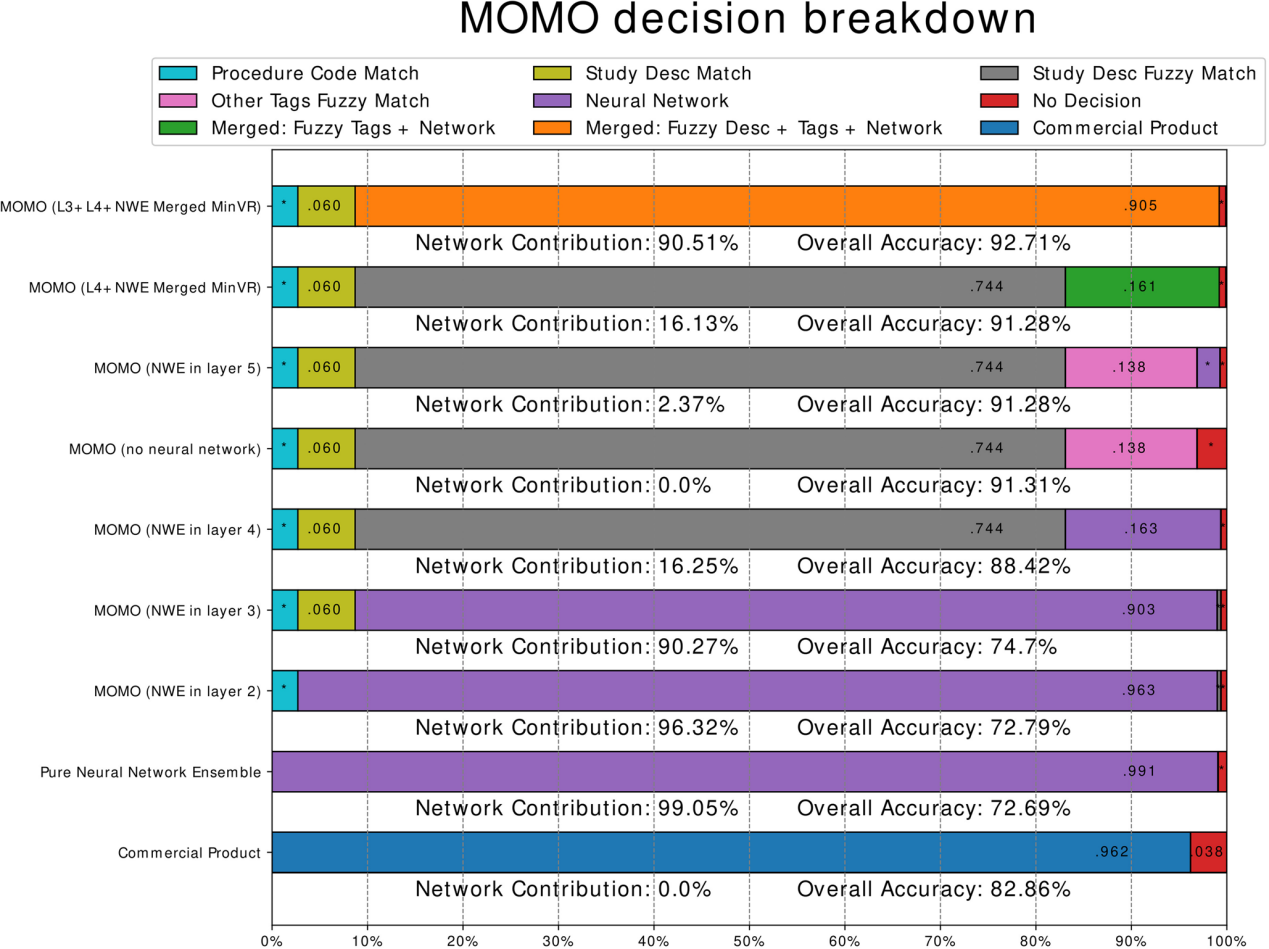
Decision breakdown. A stacked bar plot, displaying which resource MOMO used to make a decision. The algorithms are ordered as in Fig. 3. The decisions are also sorted by their layer in the algorithm. For example, if the “Procedure Code Match” was checked first in an algorithm, it appears on the left end of the bar for that algorithm

Figure 4 shows a breakdown of which information is responsible for the predictions for different variations of MOMO. The neural network ensemble has the highest prediction power (99.05% predictive power, 90.27% predictive power if exact matches for *Procedure Code* or *Study Description* are evaluated first), followed by a fuzzy matching of the *Study Description* (74.40% predictive power if exact matches are evaluated first).

## Discussion

Different hospitals use different approaches to structure and name imaging studies. This generally necessitates manual identification of such studies before they are imported into local databases. In this study, we presented MOMO, a metadata-driven algorithm, supported by an ensemble of deep neural networks. It was trained to recognize body regions and automatically classify studies. We found that the network ensemble alone can perform similarly to a commercial product, while MOMO outperforms both. We found that the neural networks offered a boost in predictive power and the best performance in our task was achieved with a variant of MOMO utilizing neural networks.

The state of the art for comparable single series prediction is given by Dratsch et al [7] for plain radiographs and Raffy et al [8] for CT and MRI, while no Deep Learning body-region classification exists for ultrasound or X-ray angiography. Our training results compare favorably to the SOTA for radiographs. For CT and MRI, our accuracies are comparatively lower, but remain fairly competitive. There are multiple explanations for this. The training set in [8] covered a larger domain (multiple hospitals, scanner types, etc.) and contained more images, likely allowing for better generalization. Beyond that, as our labels come from the PACS and not manual labeling, some labels may either be false (an abdomen study also containing a series of the chest, which was subsequently labeled “abdomen”) or correct but difficult to identify (such as a vessel CT, which can have examples of various body sites in its corresponding studies). Since the latter problems are intrinsic to the compositionality of the study classification problem, one could also argue that the comparison is not valid.

We observed that the network ensemble offered an improvement over no-network variants of MOMO (see Fig. 3). However, this improvement only applied if the networks were not given too much decision power or if the network and metadata votes were combined for more robustness. The best performance was achieved by an algorithm which combined all information in such a manner. Finally, we note that, qualitatively, the erroneous predictions made by MOMO have an increased tendency to be anatomically related to the truth, compared to the commercial product (for a comparison of their relative rates of minor and major errors, see Fig. 3).

This work has limitations. If many similar keywords are added to the reference database of the algorithm, it can cause false predictions to increase due to false positives. Some classes are underrepresented during training, potentially worsening generalization. Additionally, the dataset used for training is automatically labeled, which may decrease the performance of the neural networks. Similarly, the compositionality of different types of study poses problems for neural networks that learn to predict based on single series. Finally, the origin (single institution) and amount of training data are limiting factors.

In the future, the use of additional training data (both local and external data) will allow the networks to mitigate some of these limitations. Additionally, new preprocessing steps (like contrast enhancement or cropping) could be introduced. Furthermore, improvements could be made by incorporating new decision layers into MOMO or improving the string matching (by comparing string similarity between metadata and keywords using metrics such as Levenshtein distance). Another interesting approach may be to perform predictions directly at the study level, using a Multiple Instance Learning classifier, which is particularly well-suited to solving the problem of compositionality we experienced when classifying DICOM studies. Besides this, future work could explore the theoretical viability of the voting approach in controlled settings with varying degrees of fuzzy labels. Finally, we posit that improved standardization of metainformation and reporting across institutions, while difficult to establish in practice, would be the most straightforward and promising approach to solving the challenge that MOMO was developed for.

In summary, this is the first open-source study classification tool to use metadata and neural networks for its decision process. It is fully automated and covers all common radiological modalities, offering increased quality compared to a commercial product performing the same task, as well as reduced workload for the clinical staff.

## Supplementary information

ESM 1 (DOCX 256 kb)

## Abbreviations

CR: Conventional radiograph
DICOM: Digital Imaging and Communications in Medicine
MG: Mammogram
MOMO: Modality Mapping and Orchestration (the algorithm designed in this paper)
PACS: Picture Archiving and Communication Systems
XA/DSA: X-ray/digital subtraction angiography

## Declarations

### Guarantor

The scientific guarantor of this publication is Prof. Jens Kleesiek.

### Statistics and biometry

No complex statistical methods were necessary for this paper.

### Methodology

• retrospective

• technical study

• performed at one institution
