# Supplementary material for: Deep Learning–driven classification of external DICOM studies for PACS archiving
Source: Eur Radiol. 2022 Jul 5;32(12):8769–76. doi: 10.1007/s00330-022-08926-w (PMC9705446; doi:10.1007/s00330-022-08926-w)
Supplement: Supplementary file 1 — (DOCX 256 kb) [file 330_2022_8926_MOESM1_ESM.docx]

## **Supplementary Materials**

## S.1 Modification or disallowing of votes

The votes of the algorithm (each based on a different imaging series and DICOM tag combination) can be disallowed or modified according to a set of pre-specified rules found in the configuration file. Votes can be disallowed if the modality of the series does not match that of the study, or if blacklisted terms like “screenshot” or “report” exist in the *Series Description*, which indicate that the series may be non-representative of the whole study. Votes can be modified, if some compositions of series are detected. For example, if a study contains both “CT Abdomen” and “CT Thorax” labels, all votes for “CT Abdomen” and “CT Thorax” are replaced by votes for “CT Thorax+Abdomen”. This ruleset can easily be modified in the configuration files to represent different class compositions. As a rule of thumb, there exists a sweet spot of classification accuracy for a set of rules that is neither too large nor too small.

## S.2 Temperature Scaling

During inference, before the logits of the network are given to the softmax function, they are rescaled with a constant factor *T*, to minimize the expected difference between the network predicted and true probability (also known as confidence and accuracy, respectively). This process is called temperature scaling [15] and guarantees that the returned probabilities are statistically meaningful. For example, network predictions with a reported probability of 0.8 should be correct approximately 80% of the time. A network with this property is also called ”calibrated”. This behavior is desired so the network can learn to ”be uncertain”, instead of only making confident false predictions. This property later helps when network votes are evaluated because even if a network makes a false prediction, it can be mitigated if the network wasn’t certain about it. Thus, clear predictions are favored, and the overall classification accuracy improves. Additionally, one can throw out any series for which the prediction probability is very close to that of a randomly made prediction.

## S.3 Monte Carlo Experiment

As a proof of concept for network voting and temperature scaling, we performed a Monte Carlo experiment in which one million pseudostudies with 12 different pseudolabels were created. Each study was composed of a varying number of pseudoseries. For each of these, a skeleton version of our algorithm randomly determined whether it would correctly or incorrectly predict the label of the series and with what confidence. The random numbers were distributed such that the average network accuracy satisfied a specifiable value (the simulated accuracy reported on the X-axis in Supplementary Figure 1), as well as that the simulated network confidence was calibrated to conform to its actual accuracy. These conditions simulate an ideal environment (and neglect issues such as compositionality or different domains from which studies could come). The test was performed for varying simulated network accuracies and once each with uncorrelated and maximally correlated false predictions.

Supplementary Figure 1 shows the results of this simulation. The non-linear gain in accuracy through the voting mechanism is apparent, and large even for low network accuracies.

Additionally, we found that the temperature scaling improves the performance of the voting concept. To understand why, observe the performance line for 2 series per study at a network per-image-series accuracy of 50% in Supplementary Figure 1. Naively, one would expect 50% accuracy at the study level as well, as the network can predict either one or the other. However, as the network is calibrated, the predicted probability for a vote holds additional information. If the vote has a high probability, it is likely to be a good vote in practice, and a bad one for low probability. This increases our accuracy. We also see that a network which generalizes well to all test data will yield a significant study prediction accuracy even for a comparatively low single-image prediction accuracy. Due to all of this, we consider the voting approach justified (cf. Fig 1) and well-suited to our classification task.

This proof of concept was not performed using real-world data because the labels on such data would invariably contain an uncertain number of errors. Instead, we opted for an idealized scenario to showcase the full potential of the voting approach and consider the assessment on real-world data with fuzzy labels to be something for a future study to explore.

## S.4 Training Hyperparameter Choice

The neural networks were all trained using the same set of hyperparameters. These were found using a non-exhaustive manual search and may not represent the optimal solution. However, we found these parameters to converge reliably, no matter the network architecture or training set, with the accuracy not changing significantly for similar parameter settings and becoming worse (or not converging) for choices different by at least an order of magnitude. All training was performed with batches of size B = 24, a learning rate of $\lambda_{nominal}=1x10^{-4}$ and an exponential learning rate decay policy where $\lambda_{network}=\lambda_{nominal}*0.98^{epoch}$.

All last batches are dropped (as not dropping differently sized batches results in worse performance when using BatchNorm [17]), and all gradients are left entirely unclipped. A weight decay of $\omega_{decay}=1x10^{-4}$ is additionally used as a regularizer for the weights to help prevent overfitting on less well-represented features or classes in training. We perform no oversampling of underrepresented classes during training.

## Supplementary Figure 1: Monte Carlo Experiment

Plotted are classification results for simulated pseudostudies. The X-axis displays the accuracy of the simulated network’s predictions, the Y-axis displays the resulting classification accuracy of the pseudostudies. The different lines are color-coded, showing varying numbers of series per study. A full line indicates that false predictions are uncorrelated (false predictions have random false labels). A dashed line indicates that false predictions are maximally correlated (all false predictions use the same false label).
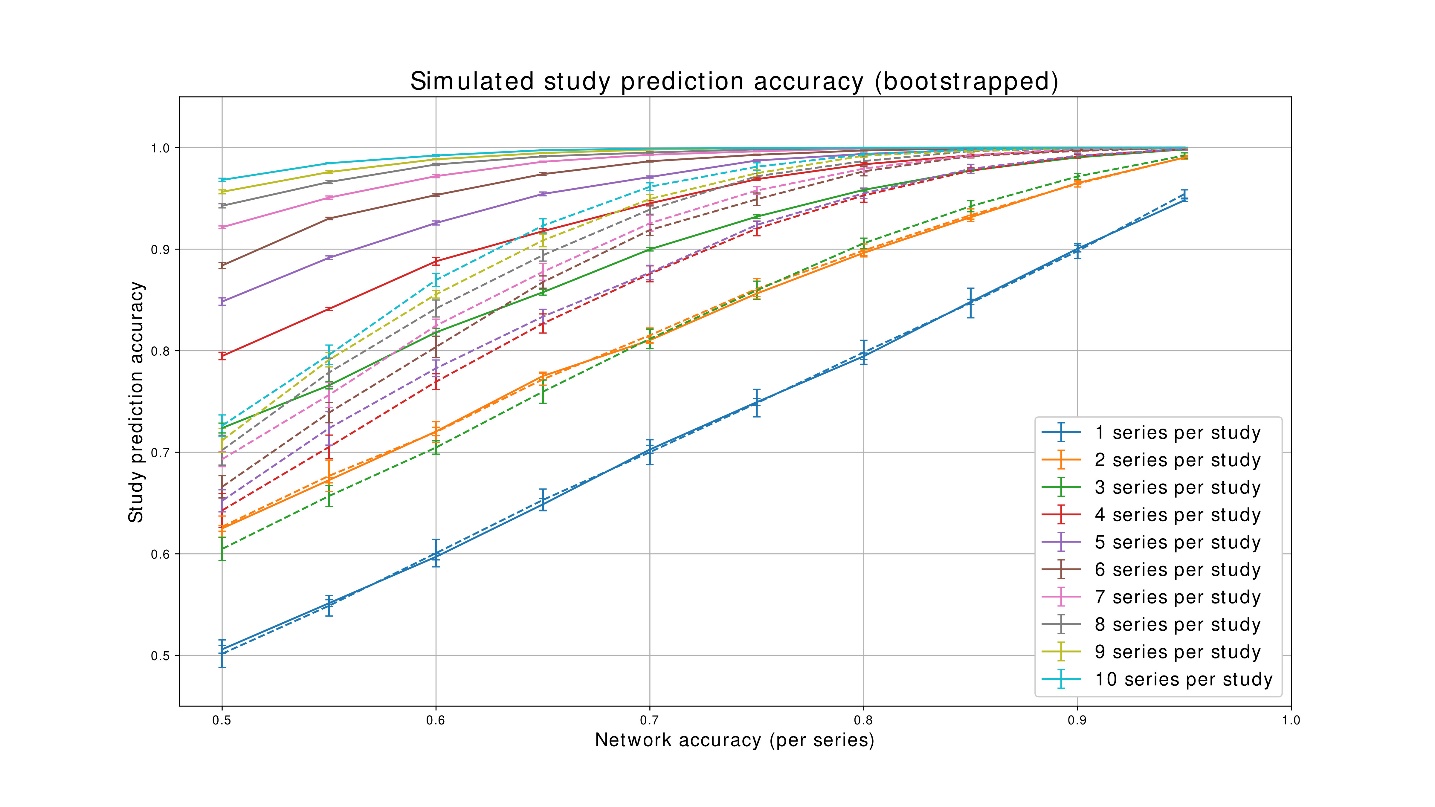


## Supplementary Table 1a: Examined Study Classes

This table gives a comprehensive list of every study class that MOMO was created to recognize.

| **Study Type** | **Modality** | **# of instances in training^d^** |
| --- | --- | --- |
| CT Abdomen | CT | 193 |
| CT Heart | CT | 171 |
| CT Lower Extremities | CT | 182 |
| CT of the Vessels | CT | 189 |
| CT Pelvis | CT | 190 |
| CT Skull | CT | 82 |
| CT Skull + Neck | CT | 150 |
| CT Spine | CT | 170 |
| CT Thorax | CT | 191 |
| CT Thorax + Abdomen | CT | 140 |
| CT Upper Extremities | CT | 197 |
| CT Whole Body | CT | 189 |
| DSA Abdomen | XA | 48 |
| DSA Angiology | XA | 15 |
| DSA Brain Vessels | XA | 0ᵃ |
| DSA Extremities | XA | 12 |
| DSA Liver | XA | 51 |
| DSA Pelvis/Upper Legs | XA | 93 |
| DSA Skull | XA | 36 |

## Supplementary Table 1b: Examined Study Classes (cont.)

| **Study Type** | **Modality** | **# of instances in training** |
| --- | --- | --- |
| DSA Skull/Neck-Intervention | XA | 16 |
| DSA Spine | XA | 10 |
| DSA Thorax | XA | 18 |
| Myelography | XA | 174 |
| Screening of the Abdomen | XA | 37 |
| Screening of the Extremities | XA | 90 |
| Screening of the Spine | XA | 153 |
| Screening of the Thorax | XA | 61 |
| Conventional, Abdomen | CR | 186 |
| Conv., Arm | CR | 738 |
| Conv., Cervical Spine | CR | 177 |
| Conv., Foot | CR | 574 |
| Conv., Hand | CR | 382 |
| Conv., Legs | CR | 635 |
| Conv., Lumbar Spine | CR | 186 |
| Conv., Mammae | CR | 124ᵇ |
| Conv., Pelvis | CR | 189 |
| Conv., Skull | CR | 196 |
| Conv., Spine | CR | 0ᵃ |

## Supplementary Table 1c: Examined Study Classes (cont.)

| **Study Type** | **Modality** | **# of instances in training** |
| --- | --- | --- |
| Conv., Thoracic Spine | CR | 188 |
| Conv., Thorax | CR | 185 |
| Mammography | MG | 124ᵇ |
| MRI Abdomen | MRI | 136 |
| MRI Cervical Spine | MRI | 157 |
| MRI Lower Extremities | MRI | 156 |
| MRI Lumbar Spine | MRI | 165 |
| MRI Mammae | MRI | 200 |
| MRI Pelvis | MRI | 194 |
| MRI Skull | MRI | 194 |
| MRI Skull + Neck | MRI | 198 |
| MRI Spine | MRI | 105 |
| MRI Thoracic Spine | MRI | 184 |
| MRI Thorax | MRI | 187 |
| MRI Upper Extremities | MRI | 144 |
| MRI Whole Body | MRI | 90 |
| PET CT Heart | PET + CT | 179ᶜ |
| PET CT Lower Extremities | PET + CT | 172ᶜ |
| PET CT Skull | PET + CT | 190ᶜ |

## Supplementary Table 1d: Examined Study Classes (cont.)

| **Study Type** | **Modality** | **# of instances in training** |
| --- | --- | --- |
| PET CT Skull + Neck | PET + CT | 166ᶜ |
| PET CT Upper Extremities | PET + CT | 55ᶜ |
| PET CT Whole Body | PET + CT | 189ᶜ |
| PET MRI Abdomen | PET + MRI | 178ᶜ |
| PET MRI Lower Extremities | PET + MRI | 160ᶜ |
| PET MRI Mammae | PET + MRI | 181ᶜ |
| PET MRI Skull | PET + MRI | 192ᶜ |
| PET MRI Skull+Neck | PET + MRI | 198ᶜ |
| PET MRI Spine | PET + MRI | 179ᶜ |
| PET MRI Thorax | PET + MRI | 198ᶜ |
| PET MRI Upper Extremities | PET + MRI | 186ᶜ |
| PET MRI Whole Body | PET + MRI | 187ᶜ |
| Ultrasound Abdomen | US | 199 |
| Ultrasound Extremities | US | 112 |
| Ultrasound Mammae | US | 83 |
| Ultrasound Neck | US | 111 |
| Ultrasound Skull | US | 5 |
| Ultrasound Thorax | US | 41 |
| Ultrasound Whole Body | US | 45 |

a) Note that some classes are not represented in the neural network training. These classes are only predicted by the metadata-driven part of the algorithm.

b) Mammographies themselves were not used to train a neural network (as there is only one single study type for the modality). They were used to train the CR-network, by labeling them as conventional radiographs of the mammae (which is what they are, ostensibly). No study not marked as modality MG (Mammography) and containing an image of the mammae exists in any of our datasets (the modality of a study being a very reliable piece of metadata), so the point is purely technical.

c) Note that the modalities which contain PET images do not have a dedicated network. In the presence of a PET image, the MRI or CT network predicts the class as if there were no PET images and then picks the corresponding PET class as answer. Thus, the non-PET images of PET+MRI or PET+CT simply become part of the MRI or CT training sets.

d) The number of instances for training varies significantly between classes (0-635). This deviation from the desired number of 200 imaging series per class has several reasons.

Firstly, not every class had enough examples in our database. Secondly, some imaging series were unavailable due to technical difficulties with the PACS database. After confirming that the neural networks performed very well, despite the imbalance of some classes, we decided to continue our project with the images we had gathered up to that point. In a sense, this makes our data a “convenience” sample. However, it importantly is 1) unbiased and 2) the developed algorithm performs quite well with the data available.

Some classes had more than 200 instances. In these cases, the classes they belong to are compositional in our database, meaning they span more than one anatomical region. We decided that for 2D images, which offer less information (leading to worse generalization), that we would try to collect 200 instances for each of these different anatomical regions (e.g. hands and wrists).

## Supplementary Table 2a: List of minor error combinations

This table gives a comprehensive list of all combinations of true study class and false predictions which would be treated as a minor mistake in our evaluation.

| **True study class** | **Misclassification as X treated as minor** |
| --- | --- |
| CT Abdomen | CT Thorax+Abdomen |
| CT Thorax | CT Thorax+Abdomen |
| CT Skull | CT Skull+Neck |
| DSA Abdomen | Screening of the Abdomen |
| DSA Brain Vessels | DSA Skull, DSA Skull/Neck-Intervention |
| DSA Extremities | Screening of the Extremities |
| DSA Liver | DSA Abdomen |
| DSA Spine | Screening of the Spine |
| DSA Skull | DSA Brain Vessels, DSA Skull-Neck Intervention |
| DSA Skull/Neck-Intervention | DSA Brain Vessels, DSA Skull |
| DSA Thorax | Screening of the Thorax |
| Screening of the Abdomen | DSA Abdomen |
| Screening of the Extremities | DSA Extremities |
| Screening of the Spine | DSA Spine |
| Screening of the Thorax | DSA Thorax |
| Conventional, Cervical Spine | Conv., Spine |
| Conv., Lumbar Spine | Conv., Spine |
| Conv., Thoracic Spine | Conv., Spine |

## Supplementary Table 2b: List of minor error combinations (cont.)

| **True study class** | **Misclassification as X treated as minor** |
| --- | --- |
| Conv., Mammae | Mammogram |
| PET CT Skull | PET CT Skull+Neck |
| PET MRI Skull | PET MRI Skull+Neck |
| MRI Cervical Spine | MRI Spine |
| MRI Lumbar Spine | MRI Spine |
| MRI Thoracic Spine | MRI Spine |
| MRI Skull | MRI Skull+Neck |

The thought process behind these minor errors is that it needs to cause no risk of additional but unnecessary examinations, to be classified as minor. If a doctor opened a patient’s files and saw “MRI Spine”, they would check if their requested lumbar spine MRI images are in that study. They might not even bother to check if it was labeled “MRI Skull” instead.

## Supplementary material references

17. Ioffe S, Szegedy C (2015) Batch normalization: accelerating deep network training by reducing internal covariate shift. In: Bach F, Blei D (eds) Proceedings of the 32nd international conference on machine learning. PMLR, pp 448–456
